# The American Medical Association and Dr. Morton

**Published:** 1864-11

**Authors:** 


### THE AMERICAN MEDICAL ASSOCIATION AND DR. MORTON.

The American Medical Association at its late session in New York adopted by nearly a unanimous vote, the following preamble and resolutions offered by Dr. Henry D. Noyes, delegate from the New York Eye Infirmary, and supported by Dr. Mauran, of Providence, R. I.:—

*Whereas,* There is now pending in Congress an appropriation donating to Dr. T. G. Morton, of Boston, the sum of $200,000 for his services in connection with the introduction of sulphuric ether as an anæsthetic agent; and

*Whereas,* The said Dr. Morton, by suits against charitable medical institutions for infringements of an alleged patent covering not only sulphuric ether, but the state of anæsthesia however produced, has placed himself beyond the pale of an honorable profession and of true laborers in the cause of science and humanity;

*Resolved,* That the American Medical Association enter their protest against any appropriation to the said Dr. Morton, because of his unworthy conduct, also because of his unwarrantable assumption of a patentable right to anæsthesia, and further, because private benificence in Boston, New York, Philadelphia and other places, has already sufficiently rewarded him for any claims which he can justly urge.

*Resolved,* That a copy of these resolutions be sent to the Chairman of the Committee of Ways and Means of the House of Representatives at Washington. Adopted.

